# Cystic Fibrosis Transmembrane Conductance Regulator (CFTR) Allelic Variants Relate to Shifts in Faecal Microbiota of Cystic Fibrosis Patients

**DOI:** 10.1371/journal.pone.0061176

**Published:** 2013-04-17

**Authors:** Serena Schippa, Valerio Iebba, Floriana Santangelo, Antonella Gagliardi, Riccardo Valerio De Biase, Antonella Stamato, Serenella Bertasi, Marco Lucarelli, Maria Pia Conte, Serena Quattrucci

**Affiliations:** 1 Public Health and Infectious Diseases Department, Microbiology Unit, ‘Sapienza’ University of Rome, Rome, Italy; 2 Regional Cystic Fibrosis Centre, Paediatrics and Infant Neuropsychiatry Department, ‘Sapienza’ University of Rome, Rome, Italy; 3 Department of Haematology and Cellular Biotechnologies, Sapienza University of Rome, Rome, Italy; Johns Hopkins School of Medicine, United States of America

## Abstract

**Introduction:**

In this study we investigated the effects of the Cystic Fibrosis Transmembrane conductance Regulator (*CFTR*) gene variants on the composition of faecal microbiota, in patients affected by Cystic Fibrosis (CF). *CFTR* mutations (F508del is the most common) lead to a decreased secretion of chloride/water, and to mucus sticky secretions, in pancreas, respiratory and gastrointestinal tracts. Intestinal manifestations are underestimated in CF, leading to ileum meconium at birth, or small bowel bacterial overgrowth in adult age.

**Methods:**

Thirty-six CF patients, fasting and under no-antibiotic treatment, were *CFTR* genotyped on both alleles. Faecal samples were subjected to molecular microbial profiling through Temporal Temperature Gradient Electrophoresis and species-specific PCR. Ecological parameters and multivariate algorithms were employed to find out if *CFTR* variants could be related to the microbiota structure.

**Results:**

Patients were classified by two different criteria: 1) presence/absence of F508del mutation; 2) disease severity in heterozygous and homozygous F508del patients. We found that homozygous-F508del and severe CF patients exhibited an enhanced dysbiotic faecal microbiota composition, even within the CF cohort itself, with higher biodiversity and evenness. We also found, by species-specific PCR, that potentially harmful species (*Escherichia coli* and *Eubacterium biforme*) were abundant in homozygous-F508del and severe CF patients, while beneficial species (*Faecalibacterium prausnitzii*, *Bifidobacterium spp.*, and *Eubacterium limosum*) were reduced.

**Conclusions:**

This is the first report that establishes a link among *CFTR* variants and shifts in faecal microbiota, opening the way to studies that perceive CF as a ‘systemic disease’, linking the lung and the gut in a joined axis.

## Introduction

Cystic fibrosis (CF; OMIM 219700) is an autosomal recessive disorder affecting the exocrine glands of the respiratory, digestive and reproductive systems. The clinical manifestations of CF in affected individuals vary widely, with both age at diagnosis and lethality ranging from the first year of life to the third (and later) decade [1]. In CF, the chronic infection of respiratory tract leads to progressive respiratory deficiency [2]. Mutations in the cystic fibrosis transmembrane conductance regulator (*CFTR*) gene lead to the accumulation of mucus on epithelial surfaces mainly in pancreas, respiratory and gastrointestinal tracts [3], [4]. The *CFTR* gene (OMIM 602421) functions as a chloride channel that regulates ions and water transport across epithelial cell membranes. Around 1500 mutations and 300 polymorphisms in the *CFTR* gene are known (Cystic Fibrosis Mutation Database; http://www.genet.sickkids.on.ca/cftr/), usually grouped in five classes representative of all the possible alterations in the maturation process and/or transfer of the CFTR protein [1], [5]. In this five-class system, mutations belonging to classes I, II, and III are predicted to have severe functional consequences on CFTR function via different molecular mechanisms. Mutations belonging to classes IV and V, on the other hand, are expected to confer some residual function to CFTR channel, and thus to give milder symptoms. Based on the residual functionality of the protein, mutations are classified as mild or severe, even if a direct correlation among *CFTR* genotype and severity of clinical manifestations is known only for the pancreatic status, not for the lung status [4]. In the gastrointestinal (GI) tract, the loss of the CFTR function results in a dehydrated state of the lumen that is believed to contribute to the insolubility of secreted mucus and glycoproteins. The GI tract represents an additional site where the CF pathology is early manifested, with severe consequences [4], [6]. In CF patients many factors predispose to the onset of the small bowel bacterial overgrowth (SBBO) condition, characterized by a high bacterial load in the small intestine [7]. Such a condition could lead to impairment of quality of life at an early stage and also later in the lifespan of an individual. Among the many factors influencing the microbiota composition, the genetic background could act a specific role, as already assessed in other studies [8], [9]. Under this hypothesis, the present study aimed to find out a correlation between faecal microbiota composition and the *CFTR* gene mutations in a cohort of CF patients. To this end, the presence of a control cohort was not necessary, because we aimed to depict the variability in microbiota composition mainly driven by different *CFTR* alleles, related to different clinical forms of CF disease. To this purpose, we selected a cohort of 36 CF patients and classified them by means of two different criteria: 1) presence/absence of F508del mutation; 2) disease severity in heterozygous and homozygous F508del patients. Dominant microbiota of faecal samples was characterized by Temporal Temperature Gradient Gel Electrophoresis (TTGE), a semi-quantitative technique useful for comparative purposes, and by species-specific PCR of cultivable bacterial species usually found in faeces. The results obtained were correlated to the two CF patients classifications, through statistical analysis, in order to find out if mutations in the *CFTR* gene could be related to the microbiota population structure. We also described shifts in faecal microbiota composition driven by *CFTR* genetic background in terms of ecological structure.

## Materials and Methods

### Ethics Statement

Faecal samples were obtained from hospitalized patients and external controls within the ‘Cystic Fibrosis Centre’ of the Department of Paediatrics at Hospital ‘Policlinico Umberto I’ of Rome. Ethics approval for this study was granted by the Ethics Committees of the ‘Sapienza’ University and ‘Policlinico Umberto I’ Hospital, Italy. Written informed consent was obtained from parents of all subjects enrolled in this study who were under 18 years of age, while the written informed consent was given autonomously by subjects over (or equal to) 18 years. In any case, written informed consent was obtained upon instructions on ethics, aims, and methodologies employed in the study.

### Patients and Classification Criteria

Thirty-six CF patients referred to the ‘Regional Cystic Fibrosis Centre’ of the Department of Paediatrics at Hospital ‘Policlinico Umberto I’ of Rome, were enrolled after written informed consent (Table 1). The study protocol was approved by the Committee on Ethical Practice of the ‘Policlinico Umberto I’ hospital. All patients enrolled did not received antibiotics during 2 month prior to the beginning of the study: this was a rigorous selection criterion, due to the difficulties in not providing antibiotics to CF patients, who usually undergo such a therapy. All patients underwent the same antibiotic treatment (200 mg of ciprofloxacin every 12 hours), suspended 2 months before faecal sampling. All patients enrolled did not suffer from ileum meconium in early stage of life. Furthermore, all patients were fasting one day prior the faecal sampling. All these criteria were chosen to homogenise and reduce the internal variability of microbiota response, in order to have a ‘common background’ to investigate the role of CFTR genetics on microbiota composition itself. The diagnostic work up of CF was according to international protocols.

**Table 1:** Patients’ genetics and demographics.

Patient	Sex	Age (years)	CFTR allele, ♂	CFTR allele, ♀	Criterion I^(a)^	Criterion II(1 = severe, 0 = mild)^(b)^	Pancreatic status^(d)^	FEV1%	BMI
1	M	17	F508del	M1V	2	(1)	1	65	17.91
2	F	23	F508del	Y569D	2	(1)	0	97	18.66
3 (s1)^(c)^	F	20	P1013L	F508del	2	(0)	0	87	18.67
4	M	11	F508del	L997F (without R117L)	2	0	0	110	21.33
5 (s1)^(c)^	M	11	P1013L	F508del	2	(0)	0	100	23.14
6	M	8	R553X	F508del	2	1	0	80	15.87
7	M	3	F508del	unknown	2	(0)	0	nd	nd
8	F	33	F508del	F508del	1	1	1	73	18.61
9	M	10	F508del	L1077P	2	1	0	94	19.79
10	M	9	F508del	G542X	2	1	1	100	16.00
11	F	9	4167delCTAAGCC	L1065P	3	nd	1	76	14.57
12	F	14	R117C (without (TG)_12_T_5_)	F508del	2	0	0	94	18.44
13	F	11	F508del	991del5	2	1	1	109	17.80
14	M	42	(TG)_12_T_5_	F508del	2	0	0	106	23.78
15 (s2)^(c)^	M	9	F508del	F508del	1	1	1	82	15.45
16	M	10	F508del	R347P	2	(0)	0	89	15.91
17 (s2)^(c)^	F	6	F508del	F508del	1	1	1	110	15.20
18 (s3)^(c)^	M	39	2789+5G>A	N1303K	3	nd	0	105	19.33
19 (s3)^(c)^	F	41	2789+5G>A	N1303K	3	nd	0	80	19.47
20	F	26	N1303K	W1282X	3	nd	1	90	19.57
21	M	7	*CFTR*dele2,3 (21 kb)	N1303K	3	nd	1	107	12.85
22	F	9	F508del	L997F (without R117L)	2	0	0	113	25.21
23	M	7	P5L	W1282X	3	nd	0	89	22.31
24	M	9	2789+5G>A	F508del	2	(1)	1	97	15.60
25	F	2	F508del	F508del	1	1	1	nd	nd
26	F	32	N1303K	N1303K	3	nd	1	107	21.22
27	M	14	L1065R	T338I	3	nd	0	116	21.50
28	M	12	711+3A>G	S549R(A>C)	3	nd	0	97	20.00
29	M	13	unknown	R117H (without (TG)_12_T_5_)	3	nd	0	104	19.36
30	M	14	F508del	G542X	2	1	1	84	21.87
31	F	13	F508del	F508del	1	1	1	85	18.00
32	F	41	2789+5G>A	N1303K	3	nd	1	84	21.08
33	F	21	L1065P	F508del	2	(0)	0	62	18.29
34	F	50	D1152H	F508del	2	(0)	0	63	23.74
35	M	29	F508del	2790-2A>G	2	(1)	0	92	24.46
36	F	45	unknown	W1282X	3	nd	0	69	23.42

a (Hm = 1; Ht = 2; N = 3).

b when the value is indicated in parenthesis tentative data are available.

c s1, s2 a s3 denote a sibling relationship for these three couples of patients.

d 1 = pancreas insufficiency, 0 = pancreas sufficiency.

### Genetic, Biochemical and Clinical Characterization of CF Patients

For all patients a mutational analysis was performed. A first-level genetic investigation was achieved by an ABI PRISM 3100 Avant genetic analyser (Applied Biosystems), through the multiplex DNA test ‘PCR/OLA/SCS’ (Brinson et al., 1997; Grossman et al., 1994), encompassing the 32 most frequent mutations of the CFTR gene. All patients who had not been characterized by PCR/OLA/SCS were subjected to a second-level genetic investigation, by an ABI PRISM 3130 xl genetic analyser (Applied Biosystems), with direct sequencing of the CFTR gene [10]. The effect of CFTR mutations found and, whenever possible, the functional class, were deduced from literature data [1]. In order to classify the patients we used as criteria of severity: 1) patients characterized by the presence of severe mutations (if known, of class I, II, III) in homozygous or compound heterozygous state and pancreas insufficiency (severe, annotated as 1 in Table 1); 2) patients characterized by the presence of mild mutations (if known, of class IV and V) in homozygous or compound heterozygous state and pancreatic sufficiency (mild, annotated as 0 in Table 1). The mutations found within this case series may be classified as follows. Class I, II or III: G542X, W1282X, F508del, N1303K, L1065P, L1077P, Y569D, S549R(A>C). Class IV or V: R117H, 2789+5G>A, TG12T5, R347P, D1152H, R117C. For the growth evaluation, for patients from birth to 20 years, the weight and height percentiles, the IBW (weigh for height index), and the Body Mass Index (BMI) were assessed, as valid criteria to give assessment of patients nutritional status for all patients age [11]. The assessment of body weight is easy to perform and very useful because the weight gain may be compromised in patients with CF. The height is generally less frequently compromised than the body weight, so its deficit is indicative of a serious and persistent malnutrition. Lung function was assessed by the quantity of air exhaled in one second during a forced breath (FEV1), expressed as a percentage of the theoretical value [12]. As a tool for assessing spirometry was used the Sensor Medics Vmax229. The sweat test was performed with the method of Gibson and Cooke pilocarpine iontophoresis [13], by using Macroduct Collection System and Chloride Meter Jenway System for lecture. The test is considered positive for Cl concentrations above 60 mmol/L, considered negative with values below 40 mmol/L (30 mmol/L for infants). The sweat test with values ranging between 40–60 mmol/L (30–60 mmol/L for infants) were considered borderline. The faecal elastase assay, ELISA stool test Schebo Pancreatic Elastase 1, was used to evaluate the pancreatic exocrine function. Faecal elastase values were considered normal in the range 200–500 mg/g of faeces, borderline between 100–200 mg/g, pathological if less than 100 mg/g of faeces.

**Table 2:** Group- and species-specific primers based on 16S rRNA sequences.

Target bacteria	Primer	Sequence (5′ to 3′)	Product size (bp)
*Bacteroides fragilis* group	g-Bfra-F	ATAGCCTTTCGAAAGRAAGAT	501
	g-Bfra-R	CCAGTATCAACTGCAATTTTA	
*Prevotella* group	g-Prevo-F	CACRGTAAACGATGGATGCC	529
	g-Prevo-R	GGTCGGGTTGCAGACC	
*Bifidobacterium* group	g-Bifid-F	CTCCTGGAAACGGGTGG	563
	g-Bifid-R	GGTGTTCTTCCCGATATCTACA	
*Clostridium coccoides* group	g-Ccoc-F	AAATGACGGTACCTGACTAA	441
	g-Ccoc-R	CTTTGAGTTTCATTCTTGCGAA	
*Bifidobacterium adolescentis*	BIA-1	GGAAAGATTCTATCGGTATGG	244
	BIA-2	CTCCCAGTCAAAAGCGGTT	
*Bifidobacterium longum*	BIL-1	GTTCCCGACGGTCGTAGAG	153
	BIL-2	GTGAGTTCCCGGCATAATCC	
*Eubacterium biforme*	EBI-1	GCTAAGGCCATGAACATGGA	463
	EBI-2	GCCGTCCTCTTCTGTTCTC	
*Eubacterium limosum*	ELI-1	GGCTTGCTGGACAAATACTG	274
	ELI-2	CTAGGCTCGTCAGAAGGATG	
*Fusobacterium prausnitzii*	FPR-1	AGATGGCCTCGCGTCCGA	199
	FPR-2	CCGAAGACCTTCTTCCTCC	
*Peptostreptococcus productus*	PSP-1	AACTCCGGTGGTATCAGATG	268
	PSP-2	GGGGCTTCTGAGTCAGGTA	
*Lactobacillus acidophilus*	LAA-1	CATCCAGTGCAAACCTAAGAG	286
	LAA-2	GATCCGCTTGCCTTCGCA	
*Escherichia coli*	ECO-1	GACCTCGGTTTAGTTCACAGA	585
	ECO-2	CACACGCTGACGCTGACCA	
*Bacteroides thetaiotaomicron*	BT-1	GGCAGCATTTCAGTTTGCTTG	423
	BT-2	GGTACATACAAAATTCCACACGT	
*Bacteroides vulgatus*	BV-1	GCATCATGAGTCCGCATGTTC	287
	BV-2	TCCATACCCGACTTTATTCCTT	
*Bacteroides distasonis*	BD-1	GTCGGACTAATACCGCATGAA	273
	BD-2	TTACGATCCATAGAACCTTCAT	
*Clostridium clostridiiforme*	CC-1	CCGCATGGCAGTGTGTGAAA	255
	CC-2	CTGCTGATAGAGCTTTACATA	

### Faecal DNA Extraction

Faecal samples were taken from fasting CF patients in a sterile environment placed into the ‘Policlinico Umberto I’ hospital, directly collected in a 500 mL sterile bottle with large neck, transferred to a 50 mL sterile tube, and immediately frozen at −80°C. Total DNA was extracted within 1 hour from sampling by QIAmp Stool Mini Kit (QIAGEN, Hilden, Germany) following manufacturer’s instructions. Starting faecal amount was set at 500 mg, picked up from different chunks within the sample itself, in order to minimize the sampling error. Upon extraction, total DNA concentration was quantified by a NanoDrop spectrophotometer (Thermo Fisher Scientific, Wilmington, Delaware, U.S.A.) at 260 nm, along with the 260/280 ratio, and integrity checked through 1% agarose gel electrophoresis containing EtBr 0.5 µg/ml. We obtained similar DNA concentrations after kit extraction from all CF patients, as assessed by Mann-Whitney U test (*P* = 0.348).

### PCR Amplification of Bacterial Gene Coding for 16S rRNA

Universal primers GCclamp-U968 (5′-CGC CCG GGG CGC GCC CCG GGC GGG GCG GGG GCA CGG GGG GAA CGC GAA GAA CCT TAC-3′) and L1401 (5′-GCG TGT GTA CAA GAC CC-3′) were used to amplify the V6–V8 region of bacterial gene coding for 16S rRNA [14]. PCR was performed with Taq DNA-polymerase (Hot Star Taq Plus, QIAGEN). PCR reaction (25 µL) contained 1× buffer per PCR, 2.5 mM MgCl_2_, 200 µM for each dNTP, 0.5 µM of GCclamp-U968 and L1401 primers, 1.25 U of Taq polymerase and 100 ng of total DNA. The samples were amplified under the following conditions: 95°C for 5 min, cycles at 94°C for 45″, 53°C for 45″, 72°C for one min and 10″, and a final step of 72°C for 30 min. This final elongation step was employed to minimize artefactual ‘double bands’, which could lead to an overestimation of the actual bacterial diversity. To rule out unspecific products a ‘touchdown PCR’ was performed with a starting annealing temperature of 58°C and decreasing it by 0.5°C each cycle to reach 53°C, followed by 30 cycles at 53°C. In order to minimize the PCR bias, three ‘touchdown PCR’ reactions were performed for each sample and subsequently pooled. To minimize hetero-duplex formation and single-stranded DNA (ssDNA) contamination during PCR amplification, that might cause sequence heterogeneity in a single TTGE band, 5 additional cycles of ‘reconditioning PCR’ were performed, taking 1/10 of the previous pooled PCR volume as template in a new reaction. In order to minimize the PCR bias, three ‘reconditioning PCR’ reaction were done for each sample and subsequently pooled for TTGE experiments. Successful reaction and DNA concentration was quantified by spectrophotometer measurements at 260 nm and DNA integrity checked through 1% agarose gel electrophoresis containing EtBr 0.5 µg/ml. 500 ng of DNA of PCR product from each sample was used to perform the subsequent TTGE experiments.

### TTGE Analysis of Amplified 16S rRNA V6-V8 Region

The DCode Universal Mutation Detection System was used for sequence-specific separation of PCR products. Electrophoresis was performed through a 1 mm thick, 16×16 cm 8% polyacrylamide gel, 7 M urea, 1.25×TAE (Tris-Acetic acid-EDTA, ethylenediaminetetraacetic acid), and, respectively, 40 µL and 400 µL of TEMED (Tetramethylethylenediamine) and 10% ammonium persulfate, using 7 litres of 1.25×TAE as the electrophoresis buffer. Electrophoresis was run at 69 V for 18 hours with an initial temperature of 66°C and a ramp rate of 0.2°C/h. For better resolution, a pre-run of 20 V for 15 minutes were held at the beginning of electrophoresis. Each well was loaded with 500 ng of amplified DNA plus a 2X gel loading dye (0.05% bromophenol blue, 0.05% xylene cyanol, and 70% glycerol). Gels were stained in the dark by immersion for 30 minutes in a solution of 6 µg/ml of EtBr in TAE 1X, de-stained in fresh TAE buffer 1.25X for 30 minutes, and photographed with DigiDoc-It system (UVP, Cambridge, UK). In order to minimize the inter-run variance, TGGE runs were conducted in triplicate.

### Species-specific PCR of Bacterial 16S rRNA

We choose to detect, by species-specific PCR, a panel of bacterial species particularly present in human faeces as reported by different authors [15], [16]. 16S rRNA gene-targeted primers were utilized to detect them (Table 2). The PCR experiments were performed as previously described [17], with some modifications. Briefly, the PCR was done in triplicate, the starting amount of DNA was 100 ng, and the total number of cycles was 25. The three PCR amplifications for each sample were subsequently pooled, concentrated with Speed Vac (Savant, Holbrook, NY, USA) to reach a final volume approximately equal to 1/3 of the original. The unified PCR reactions were titrated using two different methods: firstly, twenty-five microliters of each concentrated PCR were loaded on a 1% agarose gel containing EtBr 0.5 µg/ml, run for 1 hour at 80 V, photographed with DigiDoc-It system (UVP, Cambridge, UK), and analysed for densitometry with Phoretix 1D software (TotalLab, Newcastle upon Tyne, United Kingdom); secondly, measure of DNA concentration was performed with NanoDrop spectrophotometer (Thermo Fisher Scientific, Wilmington, Delaware, U.S.A.) at 260 nm, using one microliter of unified PCR. DNA titration values obtained from both methods were expressed as nanograms of DNA amount per microliter of PCR. A correction for number of 16S rRNA operons within each bacterial species was done, in order to ensure a suitable inter-species data normalization and interpretation. Mann-Whitney U test was employed to assess differences in relative abundances of bacterial species. A *P* value equal or less to 0.05 was considered statistically significant.

### Statistical Methods

#### Multivariate analysis

Two supervised classification methods, Partial Least Square Discriminant Analysis (PLS-DA) and Orthogonal Projection on Latent Structure Discriminant Analysis (OPLS-DA), were performed on a presence/absence matrix of TTGE bands, derived from the overall 36 TTGE profiles. These multivariate statistical analyses were employed to test the grouping behaviour of the TTGE profiles from CF patients, accordingly to two particular classification criteria: i) F508del homozygous/heterozygous/absence, ii) disease severity upon the non-F508del allele. Three-dimensional score plots were generated on a presence/absence TTGE matrix data by means of PLS-DA/OPLS-DA algorithms implemented in Simca-P+ software (Umetrics, Umeå, Sweden), taking into account two principal components and one orthogonal component. The number of useful components was determined by five-fold cross-validation. Data were automatically mean-centred and unit-variance (UV) scaled by the statistical software. These analyses correlated a number of X variables (TTGE bands) to a set of Y variables (CF patients’ category), obtaining the covariance between X and Y. PLS-DA/OPLS-DA approaches created a predictive model, validated through Fisher’s test, useful to classify CF patients according to their faecal microbiota composition, as assessed through TTGE and species-specific PCR. A *P* value less than or equal to 0.05 was considered statistically significant. *Clustered Image Mapping (CIM).* A resuming heatmap figure, to figure out the putative *CFTR* action in shaping faecal bacterial community, was obtained by the on-line software ‘CIMminer’ (http://discover.nci.nih.gov/cimminer) based on a scaled and centred dataset of TTGE bands and their OPLS-DA coefficient loadings. Weight coefficients were computed by PLS-DA/OPLS-DA algorithm with Simca-P+ software (Umetrics) for each TTGE band (87 X-variables) on each CF patients’ classification group. X and Y variables were 2D clustered, based on Euclidean distance dissimilarity matrix and agglomeration method of Ward.

### Ecological Features

Five ecological parameters were computed for each faecal TTGE profile, taking into account the number of bands, their relative position, and their volume (in pixels): Simpson biodiversity (*Hsi*), Simpson evenness (*Esi*), carrying capacity (*Rr*), Gini coefficient of concentration (*C*), and normalized area under Pareto-Lorenz curves (*PL*0.5). Simpson biodiversity index (*Hsi*), reflecting the number and relative abundances of the bacterial species within a sample, was calculated for each TTGE lane by the equation 
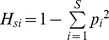
, where *p_i_* = *n_i_*/*N*, *n_i_* = volume (in pixels) of *i^th^* TTGE band, *N* = sum of total TTGE bands pixel volumes, and *S* = number of TTGE bands. Simpson evenness index (*Esi*), reflecting the distribution of species density within a sample, was calculated by the formula 
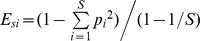
, where symbols were the same as above.

The range-weighted richness (*Rr*), reflecting the carrying capacity of the faecal habitat and richness of microbial community, was computed by the equation *Rr* = *S* ^2^*Tg, where *S* is the total number of TTGE bands in a profile and *Tg* the temperature gradient comprised between the first and the last band of the same pattern [18]. *Rr* index was subsequently normalized (*Rr_norm_*) to the value of 400, the maximum *Rr* value achievable for a faecal bacterial habitat, as found in this study and in literature [18]. Gini coefficient of concentration (*C*), reflecting the inequality of the bacterial population structure was computed by the formula 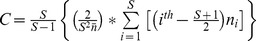, where the portion within curly braces is the Gini coefficient (*G*), *S* is the number of TTGE bands, 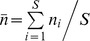, *n_i_* is the volume (in pixels) of *i^th^* TTGE band, and *i^th^* is the number of *i^th^* band (*i^th^* = 1,2,…*S*) found in a TTGE profile. Surface area between the diagonal and the Pareto-Lorenz curve (*PL*0.5), that measures the structure of the community in terms of its evenness, or the dominance of particular bacterial species, was calculated for each TTGE profile in two step. Firstly, Pareto-Lorenz curve was graphed putting on *x*-axis the cumulative normalized number of TTGE bands and on y-axis their cumulative normalized volume-intensities (in pixels). Secondly, aforementioned surface area was computed using the macro ‘area under curve’ employed in SigmaPlot 9.0 (SigmaStat), and subsequently normalized to 0.5 value (the surface area under the diagonal itself): values so obtained are comparable to the *Fo* functional organization parameter previously proposed [18]. The Mann-Whitney U test was used to assess putative differences among the ecological parameters, and a *P* value less than or equal to 0.05 was considered significant.

## Results

### Patients and CFTR Genotyping

A cohort of 36 CF patients was enrolled and underwent to *CFTR* genotyping, as described in Materials and Methods. Both maternal and paternal *CFTR* alleles were sequenced (Table 1), and prevalence of each allele in the entire CF cohort was assessed (Figure S1). The enrolled 36 CF patients had an overall of 26 different *CFTR* alleles, with a major prevalence of the mutation F508del (24/36, 66.7%), and, at a lesser extent, N1303K (6/36, 16.7%), 2789+5G>A (5/36, 13.9%) and W1282X (3/36, 8.3%), accordingly with literature (Table 1) [1], [5]. The other mutations were found in our case series to an allele frequency below 5.6%. Some of them are characteristic of certain ethnic groups, such as W1282X in the original Jews of Central Europe, 3659delC in Sweden and, to come to the Italian reality, T338I in Sardinia, 2183AA>G and R1162X in Northern Italy [1], [5]. In order to assess the influence of *CFTR* genotype on faecal microbiota, the unique cohort of patients was divided by two specific classification criteria. Following the first criterion, all the 36 CF patients were divided in three classes: Hom-F508del (homozygous F508del) (n = 5), Het-F508del (heterozygous F508del) (n = 19), and Non-F508del (absence of F508del) (n = 12). Following the second criterion, only patients with homozygosis (n = 5) or heterozygosis (n = 19) for F508del mutation were taken into account. For this second criterion, a combination of the two *CFTR* alleles plus a clinical evaluation (Forced expiratory volume - FEV1%, exocrine pancreas function, and Body mass index - BMI) were taken into account, and a final score of ‘mild’ (n = 10) and ‘severe’ (n = 14) was given to each patient (Table 1). The first criterion encompassed all 36 CF patients, whilst the second one comprehended 24 CF patients harbouring at least one F508del allele.

### F508del Mutation Drives a Different Faecal Microbiota Composition

**Figure 1:**
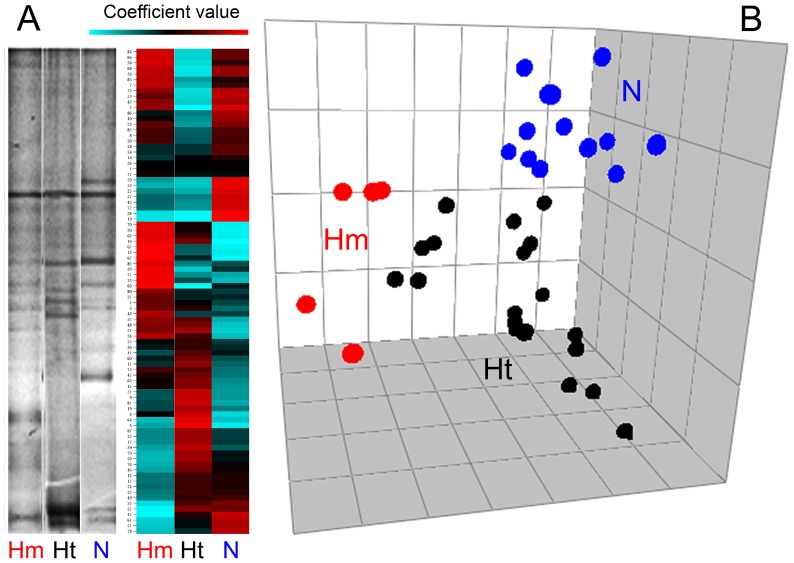
PLS-DA analysis of TTGE profiles, according to F508del mutation. Panel A) On the left are reported representative TTGE profiles from homozygous-F508del (Hm), heterozygous-F508del (Ht) and non-F508del (N) patients. Simca-P+ software was used to compute weight coefficients for each TTGE band (87 *x* variables) on the three different sub-groups of CF patients (3 *y* variables, Hm-Ht-N), with a scaled and centred data set. These coefficients were useful to interpret the influence of the x variables on the y ones. A clustered image heatmap was generated with CIMminer online software (panel A, right). As depicted in the color-coded legend, the higher that the coefficient value is, the higher the weight (red), while the lower that the value is, the lower the weight (turquoise). Panel B) Faecal TTGE profiles of all 36 CF patients were analysed by PLS-DA, and the resulting 3D score plot model gave a significant separation between the three sub-groups: homozygous-F508del (Hm, red circles); heterozygous-F508del (Ht, black circles), and non-F508del (N, blue circles).

Our cohort consisted of 26 *CFTR* genotypes (Table 1, Figure S1), allowing the examination of putative relationships existing among *CFTR* alleles and faecal bacterial community composition. Due to the high prevalence of F508del mutation (Figure S1), we choose to divide patients in 3 classes: homozygous-F508del (n = 5), heterozygous-F508del (n = 19) or non-F508del mutation (n = 12). Their TTGE profiles of faecal microbiota were regressed against this first criterion by means of PLS-DA algorithm, to assess if F508del mutation could affect the faecal microbiota structure in a particular way. We reported representative mean TTGE profiles for each of the three classes, along with a heatmap based on Euclidean distance generated by means of the online tool CIMminer (Figure 1, panel A). In this latter, the red areas correspond to the TTGE bands with higher importance in dividing the three classes, showing how the different genetic background, due to the homozygous-F508del, heterozygous-F508del and non-F508del, arise different faecal microbiota compositions in CF patients. A score plot obtained by the PLS-DA analysis was also depicted (Figure 1, panel B), showing a significant separation among the three classes of CF patients (Total error = 0%, Fisher’s *P = *3.1*10^−14^).

### Faecal Microbiota Composition is Shaped by CF Disease Severity

**Figure 2:**
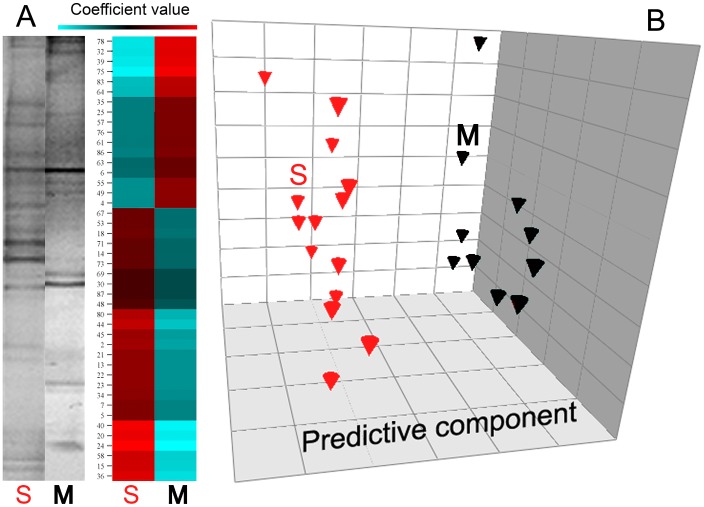
OPLS-DA analysis of TTGE profiles, according to disease severity. Panel A) On the left are reported representative TTGE profiles from severe and mild CF patients. Simca-P+ software was used to compute weight coefficients for each TTGE band (87 *x* variables) on the two subgroups of CF patients (2 *y* variables) harbouring at least one F508del mutation in one allele: severe (S) and mild (M), with a scaled a centred data set. A clustered image heatmap was generated with CIMminer online software (panel A, right). As depicted in the color-coded legend, the higher that the coefficient value is, the higher the weight (red), while the lower that the value is, the lower the weight (turquoise). Panel B) Faecal TTGE profiles of all 24 CF patients harbouring at least one F508del allele were analysed by OPLS-DA, and the resulting 3D score plot model gave a significant separation among the two sub-groups: severe (S, black diamonds) and mild (M, red diamonds).

*CFTR* gene mutations are commonly divided in 5 classes, upon their phenotypic/clinical severity [1], [5]. In order to assess if the faecal microbiota structure would be affected by CF disease severity, we choose all the CF patients harbouring at least one F508del mutation. Once established that F508del drives a different faecal microbiota composition (see above), we focused our attention on homozygous-F508del (n = 5) and heterozigous-F508del (n = 19) patients, dividing them into two distinct classes: mild (n = 10) and severe (n = 14). Their TTGE profiles of faecal microbiota were regressed against this second criterion by means of OPLS-DA algorithm: this criterion was chosen because F508del mutation was found in 24/36 (66.7%) of CF patients, thus allowing a common background to investigate the role of the other *CFTR* allele on faecal microbiota composition. We reported representative mean TTGE profiles for each of the two classes, along with a heatmap based on Euclidean distance generated by means of the online tool CIMminer (Figure 2, panel A). In the heatmap, red areas correspond to the TTGE bands with higher importance in dividing the severe and mild classes, showing how the different genetic background, due to the other non-F508del allele, could shape a different faecal microbiota compositions in CF patients. A score plot obtained by the OPLS-DA analysis was also drawn (Figure 2, panel B), showing a significant separation among the two classes of CF patients (Total error = 0%, Fisher’s *P = *1.2*10^−6^). Thus, CFTR mutations, driving a mild or severe clinical phenotype in CF patients, could also drive, in some measure, the faecal microbiota composition.

### Ecological Parameters Describe the Faecal Microbiota Structure in CF Patients

**Figure 3:**
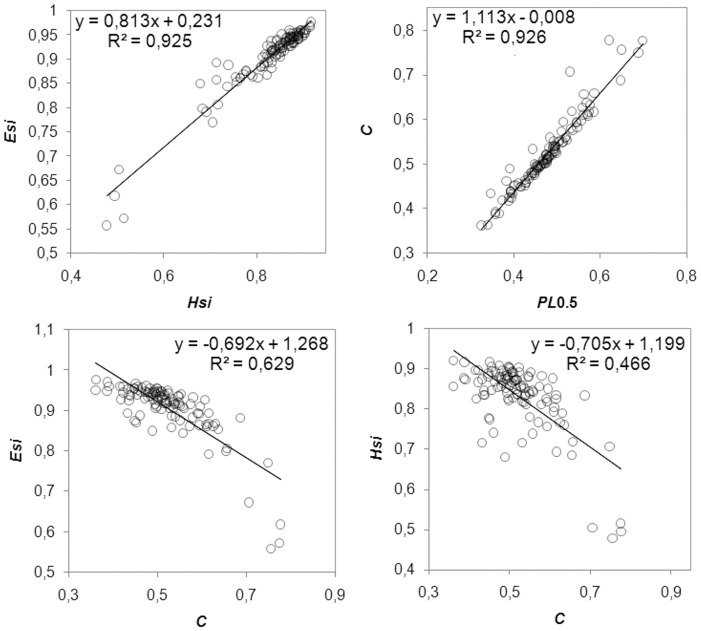
Correlations among ecological features. Faecal TTGE profiles were used to compute five ecological parameters: Simpson biodiversity (*Hsi*), Simpson equiripartition index (*Esi*), normalized carrying capacity (*Rr_norm_*), Gini coefficient of inequality (*C*), and normalized Pareto-Lorenz curves (*PL*0.5). Each panel reports the linear equation y = mx+q and the R^2^ value, to assess a positive or negative correlation and the goodness of linear fit. *Rr_norm_* gave no correlation with any of the other ecological parameters (correlation not shown).

To determine which aspects of community composition were most related to CFTR mutation, specific ecological parameters were computed for each TTGE profile: Simpson biodiversity (*Hsi*), Simpson equiripartition index (*Esi*), normalized carrying capacity (*Rr_norm_*), Gini coefficient of inequality (*C*), and normalized Pareto-Lorenz curves (*PL*0.5). Even if the TTGE is considered a semi-quantitative technique, it can describe bacteria that constitute up to 1% of the total bacterial community [19], [20]: thus, due to this resolution power, it can properly describe changes in bacterial community structure. Taking into account all CF patients, without any classification, we found linear correlations among the ecological parameters: *Esi* and *Hsi* (R^2^ = 0.925), *C* and *PL*0.5 (R^2^ = 0.926) were linearly and positive correlated, whilst *Esi* and *C*, *Hsi* and *C* were linearly correlated with a negative slope (Figure 3). *Rr_norm_* parameter showed no correlation with *Esi*, *Hsi*, *C*, or *PL*0.5 (results not shown). Mann-Whitney U test was used to calculate putative ecological differences among faecal microbiota of homozygous-F508del, heterozygous-F508del and non-F508del groups (criterion I). The same test was used to quantify differences in ecological parameters among severe and mild CF patients (criterion II). In Table 3 are the results. Homozygous F508del mutation led the faecal microbiota to higher evenness (*Esi*) (*P* = 0.027) and higher biodiversity (*Hsi*), compared to heterozygous-F508del and non-F508del. Severe CF patients showed a faecal microbiota with lesser inequality of the bacterial population structure (*C*) and lesser dominance of particular bacterial species (*PL*0.5), compared to the mild one (*P* = 0.024). At a microbial community level, these results could explain why F508del mutation led to a different faecal microbiota composition, as well as the CF disease severity did.

**Table 3:** Ecological parameters.

Parameter	Homozygous F508del	Heterozygous F508del	Non-F508del	*P* value^a^	Severe	Mild	*P* value
*Hsi*	0.863±0.008	0.820±0.011	0.829±0.016	**0.048** (Hm-Ht)	0.840±0.009	0.813±0.019	0.398
*Esi*	0.933±0.005	0.897±0.010	0.905±0.012	**0.027** (Hm-Ht)	0.917±0.007	0.888±0.017	0.112
*Rr_norm_*	0.503±0.071	0.370±0.028	0.407±0.040	**0.019** (Hm-Ht)	0.398±0.038	0.398±0.039	0.936
*C*	0.492±0.015	0.525±0.011	0.538±0.013	0.142 (Hm-N)	0.502±0.013	0.539±0.014	**0.024**
*PL*0.5	0.456±0.015	0.478±0.011	0.489±0.010	0.179 (Hm-N)	0.460±0.012	0.493±0.013	**0.024**

a Hm, homozygous F508del; Ht, heterozygous F508del; N, non-F508del. The lowest *P* value was reported for all comparisons (within round brackets is reported the corresponding match). In bold are the significant *P* values.

### Particular Bacterial Species are Related to the CF Disease Phenotype

**Figure 4:**
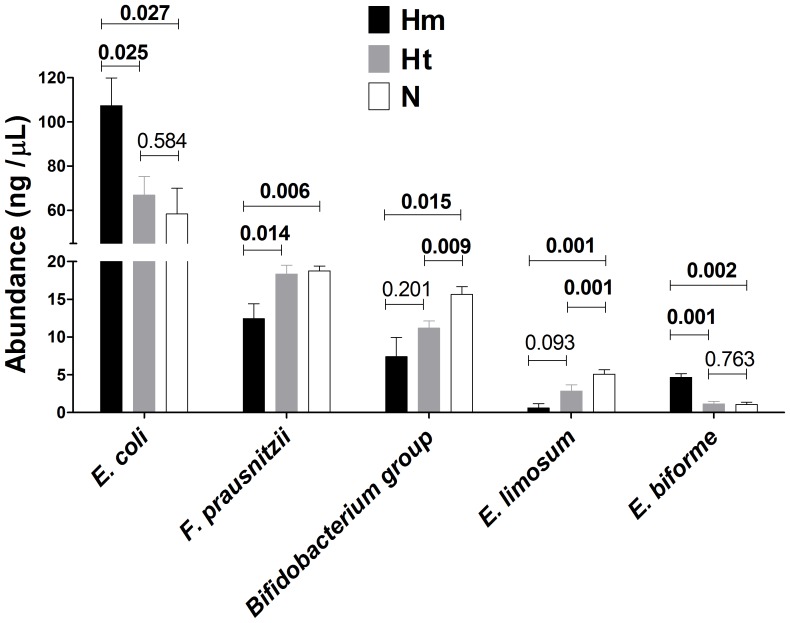
Species-specific PCR, according to F508del mutation. Species-specific PCR were performed on 16 bacterial species or groups, and a Mann-Whitney U test was employed to assess putative differences in their relative abundances (expressed as ng/µL) among homozygous-F508del (Hm, black bars), heterozygous-F508del (Ht, grey bars) and non-F508del (N, white bars) patients. In figure were reported the five bacterial species (or groups) with significant *P* values (in bold) among sub-groups.

**Figure 5:**
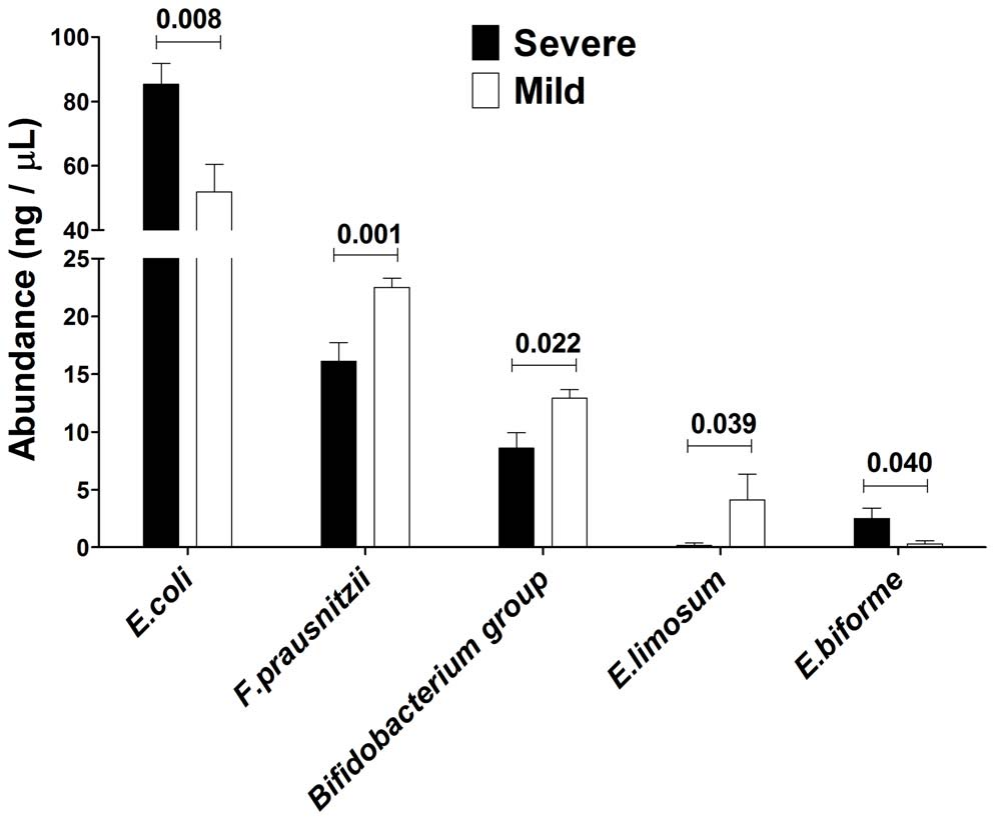
Species-specific PCR, according to disease severity. Species-specific PCR were performed on 16 bacterial species or groups, and a Mann-Whitney U test was employed to assess putative differences in their relative abundances (expressed as ng/µL) among severe (black bars) and mild (white bars) CF patients. In figure were reported the five bacterial species (or groups) with significant *P* values (in bold) among sub-groups.

Once established that F508del mutation and CF disease severity drive a particular faecal microbiota composition, showing different values of ecological parameters, we shifted our attention from the microbiota to some of its constituting bacterial species. Species-specific PCR assays were performed to achieve a presence/absence and a relative quantification of some cultivable bacterial species usually found in faeces, as reported in literature [15], [16]. After an accurate correction for the intra-species number of 16S rDNA operons, a matrix of relative quantification based on 1% agarose gel densitometry was built, combined with species-specific prevalence data, and processed with Mann-Whitney U test to assess putative differences in bacterial relative abundances. Among sixteen bacterial species or groups examined (Table 2), only five were found to be differentially distributed, in terms of relative abundance, in faecal samples of CF patients divided by the two classification criteria: *Escherichia coli*, *Faecalibacterium prausnitzii*, *Bifidobacterium* group, *Eubacterium limosum*, *Eubacterium biforme*. We found higher levels of *E. coli* and *E. biforme* in homozygous-F508del patients, and, at the same time, higher levels of *F. prausnitzii*, *Bifidobacterium*, and *E. limosum* in non-F508del patients (Figure 4). Taking into account the criterion II, we found that severe CF patients harboured more *E. coli* and *E. biforme* than mild one, which, in turn, had higher levels of *F. prausnitzii*, *Bifidobacterium*, and *E. limosum* in their faecal samples (Figure 5).

## Discussion

Recently, the intricate molecular mechanisms underpinning the host-microbe cross-talk has begun to emerge. Gut microbiota, the biggest bacterial community in our body, exerts many beneficial effects on our health, acting like an ‘organ’ with peculiar characteristics in its composition and function [21]. Many features can shape the gut microbiota structure: sex [22], diet [23], birth delivery [24], feeding mode [25], and disease status [26]. Up to date, only two studies focused on the role of human genetics as a modifier agent of gut microbiota composition: the first, in Inflammatory Bowel Diseases (IBD) [9], and the second, in Familiar Mediterranean Fever (FMF) [8]. Like FMF, Cystic fibrosis is a good candidate to assess how the genetic background could shape the gut microbiota, due to its monogenic nature (unlike IBD, where 99 loci are known to play a role [27]). During development, intestine is the first district in which high levels of CFTR mRNA are expressed, and are constitutively maintained during all the lifetime [28]. The intestine usually presents high protein or mucous loads, together with low flow rates, and CFTR defects lead to abnormalities in: gastrointestinal tissue morphology, electrolytes secretion, mucus secretion, protein concentration, and protein folding [28]. For this reason, a loss of function of CFTR protein can lead to intestinal obstruction (called ileum meconium) as the earliest clinical phenotype of CF, and to intestinal complications in paediatric/adult age as a secondary site of CF manifestations (after the lung) [28]. Recently, it was found how a diminished pH in airway epithelia from *CFTR* ^−/−^ pigs impaired bacterial killing [29], and it could be speculated a similar event also in the gastrointestinal tract, where a particular microbiota could arise [30].

Our study hypothesis was that *CFTR* genetic background could act as a selective force, able to drive a different gut microbiota, as well as the faecal one, in CF patients. Gut microbiota encompasses mucosa-associated and luminal microbiota, while faeces mainly harbor the luminal part of the gut microbiota. Therefore, we showed here how *CFTR* gene variants were related to shifts in faecal microbiota profiles of CF patients. Particularly, we found that the most common *CFTR* mutation, F508del, found in 66.7% of our patients, led to a peculiar composition of faecal microbiota, especially in the homozygous state (Fig. 1), in which *E. coli* and *E. biforme* species prevailed (Fig. 4). The potential effect that differences in sample size could have on data interpretation, e.g. in the case of the five homozygous F508del patients, was considered and taken into account in the multivariate statistical analysis by intra-variance correction. Such results are to be considered indicative for future studies addressed to investigate the role of homozygous F508del state in human subjects more deeply. F508del mutation arose around 11000 years ago, spreading out during the Neolithic human expansion, due to a putative selective advantage of heterozygous F508del state [31]. Following literature data, F508del mutation belongs to the second class of *CFTR* mutations, in which loss of function of the resulting CFTR protein heavily impairs chloride secretion, leading to a higher thickness and viscosity of the mucus layer [1], [5]. It could be arguable that such a peculiar intestinal habitat, along with an inflamed mucosal state, could enhance the adhesion capability of particular bacterial species, such as *E. coli* and *E. biforme*, leading to higher counts in their populations, as already established for *E. coli* strains in Inflammatory Bowel Diseases [32]. Indeed, we found these two bacterial species to be prevalent also in faecal samples from severe CF patients, not from mild (Fig. 5). The mean percentages of *E. coli* and *E. biforme* in faecal samples are usually 1.21% [33] and 1.00% [34], respectively. Thus, the expected *E. coli*/*E. biforme* ratio of the mean relative abundances in a faecal sample from healthy subjects should be around 1.2. Actually, in a parallel on-going study, we found that *E. coli*/*E. biforme* mean ratio within our cohort of healthy subjects was 1.6, and this ratio grew up to 50.4 in an age- and sex-matched CF cohort (unpublished data, not shown here). Notably, *E. coli* differs from *E. biforme* not only for the cell wall properties (the former is Gram-negative, the latter is Gram-positive), but also for antibiotic susceptibility [35].

*E. coli* was found to be highly prevalent in homozygous-F508del and severe CF patients, which also harboured a different faecal microbiota (Fig. 1 and 2). The high prevalence of *E. coli* found could be due to its enhanced fitness in the CF intestinal habitat as already observed in Celiac disease [36] and IBD [32], [37]. We could hypothesize that high levels of *E. coli* in homozygous F508del and severe CF patients would be related to a ‘pathobiont’ sub-population. Since there is an increasing evidence of intestinal inflammation in CF [38], this habitat could be at the origin of a positive selection for inflammation-adapted *E. coli* ‘pathobionts’, as well as it happens in other disorders. In IBD patients with underlying genetic mutations, inflammation is targeted to specific members of the microbiota and not to infectious pathogens [39]. Moreover, other authors found that *Escherichia* genus, as well as *Clostridium* and *Enterococcus*, that reside in all people, are usually targeted by T cell response in an inflamed intestinal habitat, leading to the ‘pathobiont’ hypothesis [40], [41]. *E. biforme* resulted to be significantly present in homozygous-F508del (Fig. 4) and severe CF patients (Fig. 5). This bacterial species was isolated from human faeces, rumen, sewage and soil, but it was occasionally isolated from wounds, abscesses and periodontitis, thus it may be considered an opportunistic pathogen [34], [42]. At the same time, both in homozygous-F508del and severe CF patients, we also found a marked reduction in beneficial bacterial species, as stated in literature, such as *Faecalibacterium prausnitzii* [43], *Bifidobacterium spp.*, and *Eubacterium limosum* [44] (Fig. 4 and 5). Interestingly, it is conceivable that *E. biforme* doesn’t belong to the same genus of *E. limosum*, because the former belongs to Erysipelotrichi class, while the latter belongs to Clostridia class, both within the Firmicutes phylum. Thus, a within-the-genus different bacterial habit should be taken unambiguously, albeit *E. biforme* and *E. limosum* apparently have the same ‘genus’ designation. From the aforementioned results, one could realize that *CFTR* genetic background in homozygous-F508del and severe patients led to an enhanced dysbiosis, with the prevalence of *E. coli* and *E. biforme* species, even within the CF cohort itself. Indeed, we found that biodiversity (*Hsi*), evenness (*Esi*), and carrying capacity (*Rr_norm_*) were significantly higher in homozygous-F508del patients, while inequality (C) and community dominance (*PL*0.5) were lower in severe CF patients (Table 3). These results are in agreement with the hypothesis of an enhanced SBBO in the gut of CF patients [7], eventually leading to a major load of bacterial counts and by-products that could stimulate an exaggerated mucosal response in the gut. Owing to faeces that mainly represent the luminal bacteria, in order to properly assess an *in situ* dysbiotic event, further experiments should be done on intestinal biopsies from CF patients, similarly to what has recently been done on samples from transplanted lungs [45]. Such a requirement could be difficult to be achieved, because CF patients usually don’t undergo gastro-duodenoscopy or endoscopy in their clinical follow-up, and, most importantly, intestinal symptoms are commonly underestimated [2], [6], [28]. It is noteworthy that our cohort was not under antibiotic treatment for two months before faecal sampling, nor the patients underwent a probiotic usage: thus, the role of the *CFTR* gene on shaping the faecal microbiota was not influenced by such treatments. Previously, it was demonstrated a relation among *CFTR* gene mutations and modifications induced in the airway microbiota in 51 CF patients [46], thus, the common genetic background could establish a link among the two main body districts in which CFTR mRNA is expressed: the lung and the gut [28]. Recent studies evidenced an emerging view of an intimate cross-talk [47] existing among the lung and the gut, especially in CF [48]. Thus, CF disease could be perceived as a ‘systemic disease’, in which pulmonary and intestinal habitats are interconnected in a ‘lung-gut axis’ by immunological system, common genetic background (like *CFTR*), and, ultimately, some bacterial or viral species, or they by-products [47]. Clinical studies should be pointed towards a holistic view of the comorbidities existing within a disease: such an effort should also ameliorate the CF treatment regimens. The present study was only on an observational basis: further experiments with mice, or pigs, carrying mutated *CFTR* variants, would shed light on the actual microbiota-modulating properties of *CFTR* gene. This kind of study would improve the knowledge on the refined interactions existing among host and microbiota, important step to set up new therapy strategies aimed at restoring and maintaining an healthy intestinal microbiota in CF patients, and to ameliorate their clinical conditions.

## Supporting Information

Figure S1 CFTR alleles prevalence. All *CFTR* mutations found in this study are reported in descending order of prevalence. On *x*-axis is shown the percentage of patients (n = 36, black bars), or the percentage of *CFTR* alleles (n = 72, white bars), harbouring almost one copy of the mutation depicted on *y*-axis.
(TIF)
