# Correction: Cystic Fibrosis Transmembrane Conductance Regulator (CFTR) Allelic Variants Relate to Shifts in Faecal Microbiota of Cystic Fibrosis Patients

**DOI:** 10.1371/annotation/a8b06cd3-c8a8-48fa-9748-b04fded4b963

**Published:** 2013-07-17

**Authors:** Serena Schippa, Valerio Iebba, Floriana Santangelo, Antonella Gagliardi, Riccardo Valerio De Biase, Antonella Stamato, Serenella Bertasi, Marco Lucarelli, Maria Pia Conte, Serena Quattrucci

In the Introduction section of this article, the authors used several sentences from previous publications without quoting the original sources. The authors would like to apologize for this and acknowledge the original authors of the borrowed text.

The publications involved are the following:

Kosova G, Pickrell JK, Kelley JL, McArdle PF, Shuldiner AR, et al. (2010) The CFTR Met 470 Allele Is Associated with Lower Birth Rates in Fertile Men from a Population Isolate. PLoS Genet 6(6): e1000974. doi:10.1371/journal.pgen.1000974

Norkina O, Burnett TG, De Lisle RC. Bacterial overgrowth in the cystic fibrosis transmembrane conductance regulator null mouse small intestine. Infect Immun. 2004 Oct;72(10):6040-9.

Ahmed N, Corey M, Forstner G, Zielenski J, Tsui LC, Ellis L, Tullis E, Durie P. Molecular consequences of cystic fibrosis transmembraneregulator (CFTR) gene mutations in the exocrine pancreas. Gut. 2003 Aug;52(8):1159-64.

The sentences which overlap with previous publications, and not quoted within the Introduction, were:

1) Cystic fibrosis (CF; OMIM 219700) is an autosomal recessive disorder affecting the exocrine glands of the respiratory, digestive and reproductive systems. The clinical manifestations of CF in affected individuals vary widely, with both age at diagnosis and lethality ranging from the first year of life to the third (and later) decade.

The CFTR gene (OMIM 602421) functions as a chloride channel that regulates ions and water transport across epithelial cell membranes.

Related reference: Kosova G, Pickrell JK, Kelley JL, McArdle PF, Shuldiner AR, et al. (2010) The CFTR Met 470 Allele Is Associated with Lower Birth Rates in Fertile Men from a Population Isolate. PLoS Genet 6(6): e1000974. doi:10.1371/journal.pgen.1000974

2) In the gastrointestinal (GI) tract, the loss of the CFTR function results in a dehydrated state of the lumen that is believed to contribute to the insolubility of secreted mucus and glycoproteins.

Related reference: Norkina O, Burnett TG, De Lisle RC. Bacterial overgrowth in the cystic fibrosis transmembrane conductance regulator null mouse small intestine. Infect Immun. 2004 Oct;72(10):6040-9.

3) In this five class system, mutations belonging to classes I, II, and III are predicted to have severe functional consequences on CFTR function via different molecular mechanisms. Mutations belonging to classes IV and V, on the other hand, are expected to confer some residual function to CFTR channel, and thus to give milder symptoms.

Related reference: Ahmed N, Corey M, Forstner G, Zielenski J, Tsui LC, Ellis L, Tullis E, Durie P. Molecular consequences of cystic fibrosis transmembraneregulator (CFTR) gene mutations in the exocrine pancreas. Gut. 2003 Aug;52(8):1159-64.

The identified issues have no bearing on the results and conclusions of the study. The authors apologize for the instances of plagiarism noted above. 

