# Supplementary figures and images for: Cystic Fibrosis Transmembrane Conductance Regulator (CFTR) Allelic Variants Relate to Shifts in Faecal Microbiota of Cystic Fibrosis Patients
Source: PLoS One. 2013 Apr 17;8(4):e61176. doi: 10.1371/journal.pone.0061176 (PMC3629184; doi:10.1371/journal.pone.0061176)

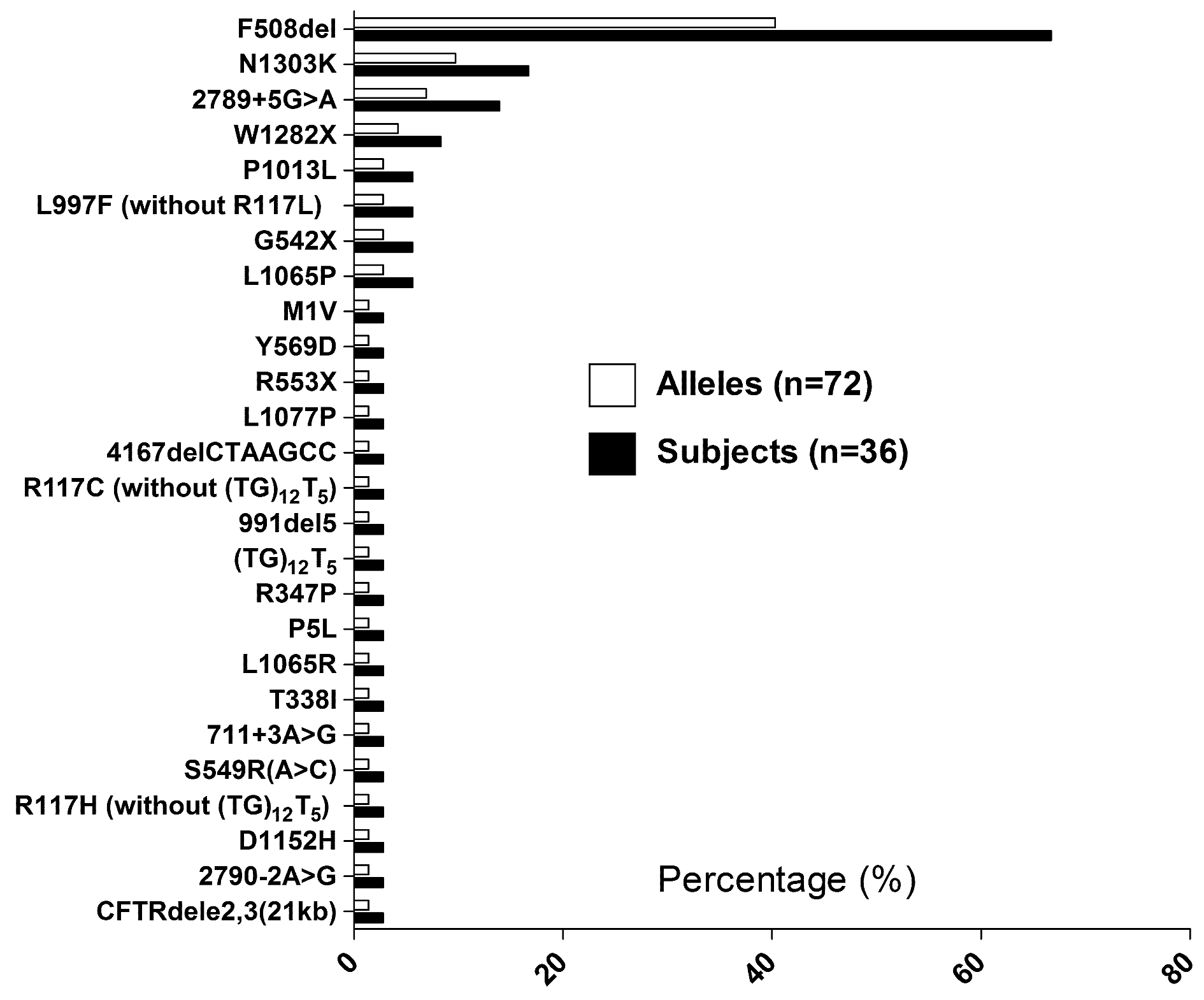

Supplement: Figure S1 — CFTR alleles prevalence. All CFTR mutations found in this study are reported in descending order of prevalence. On x-axis is shown the percentage of patients (n = 36, black bars), or the percentage of CFTR alleles (n = 72, white bars), harbouring almost one copy of the mutation depicted on y-axis. (TIF) [file pone.0061176.s001.tif]
